# Platelet derived growth factor promotes the recovery of traumatic brain injury by inhibiting endoplasmic reticulum stress and autophagy-mediated pyroptosis

**DOI:** 10.3389/fphar.2022.862324

**Published:** 2022-10-19

**Authors:** Fangfang Wu, Renkan Zhang, Weiyang Meng, Lei Liu, Yingdan Tang, Leilei Lu, Leilei Xia, Hongyu Zhang, Zhiguo Feng, Daqing Chen

**Affiliations:** ^1^ Department of Emergency, The Second Affiliated Hospital and Yuying Children’s Hospital, Wenzhou Medical University, Wenzhou, China; ^2^ The First Hospital of Jiaxing or The Affiliated Hospital of Jiaxing University, Jiaxing, China; ^3^ Department of Emergency, Wenzhou People’s Hospital, The Third Clinical Institute Affiliated to Wenzhou Medical University, Wenzhou Medical University, Wenzhou, Zhejiang, China; ^4^ School of Pharmaceutical Sciences, Wenzhou Medical University, Wenzhou, China

**Keywords:** PDGF ameliorates traumatic brain injury, PDGF, traumatic brain injury, pyroptosis, er stress, autophagy

## Abstract

Autophagy and endoplasmic reticulum stress (ER stress) are important in numerous pathological processes in traumatic brain injury (TBI). Growing evidence has indicated that pyroptosis-associated inflammasome is involved in the pathogenesis of TBI. Platelet derived growth factor (PDGF) has been reported to be as a potential therapeutic drug for neurological diseases. However, the roles of PDGF, autophagy and ER stress in pyroptosis have not been elucidated in the TBI. This study investigated the roles of ER stress and autophagy after TBI at different time points. We found that the ER stress and autophagy after TBI were inhibited, and the expressions of pyroptosis-related proteins induced by TBI, including NLRP3, Pro-Caspase1, Caspase1, GSDMD, GSDMD P30, and IL-18, were decreased upon PDGF treatment. Moreover, the rapamycin (RAPA, an autophagy activator) and tunicamycin (TM, an ER stress activator) eliminated the PDGF effect on the pyroptosis after TBI. Interestingly, the sodium 4-phenylbutyrate (4-PBA, an ER stress inhibitor) suppressed autophagy but 3-methyladenine (3-MA, an autophagy inhibitor) not for ER stress. The results revealed that PDGF improved the functional recovery after TBI, and the effects were markedly reversed by TM and RAPA. Taken together, this study provides a new insight that PDGF is a potential therapeutic strategy for enhancing the recovery of TBI.

## Introduction

Exploring the knowledge of the pathophysiology after traumatic brain injury (TBI) is very necessary for better and patient-oriented treatment (Garvin and Mangat, 2017; Srivastava and Cox, 2018; Zeiler et al., 2021). Since the primary insult represents the direct mechanical damage and cannot be therapeutically influenced, the treatment aim is mainly to limit the secondary damage (Butterfield and Reed, 2016). Previous studies have shown that endoplasmic reticulum (ER) stress and autophagy are critical mechanisms involved in secondary injury post-TBI (Sun et al., 2016; Yin et al., 2017; Gao et al., 2018; Wang et al., 2019). It was reported that ER stress and autophagy were actived after TBI (Yin et al., 2017). The communication between the ER stress and autophagy is essential for cellular homeostasis and its disruption play a key role in the pathological process of TBI (Arruri and Raghu, 2022; Yin et al., 2017). Therefore, it’s a potential strategy by targeting ER stress and autophagy for TBI treatment.

As is well known, ER stress is induced by the accumulation of misfolded/unfolded proteins (Hughes and Mallucci, 2019). Autophagy is a process that can degrade damaged cytoplasmic proteins and ageing organelles, which mostly relies on the lysosomal pathway (Yin et al., 2017). Studies reported that dysregulation of ER stress and autophagy will affect cell survival and tissue repair (Annadurai et al., 2020). For instance, the blockage of ER stress or autophagy attenuated TBI-induced traumatic damage and functional outcomes (Wang D. Y. et al., 2021). Other study suggested that IL-33 alleviated TBI-induced brain edema through inhibiting ER stress and autophagy (Gao et al., 2018). Our previous study suggested that ER stress inhibition promoted microtubule assembly, and stabilized the microtubule after TBI (Wu et al., 2019). In addition, it has been revealed that inflammasome-mediated pyroptosis was also involved in the pathogenesis of TBI. Ge et al. (2018) reported that the NLRs and AIM2 inflammasome-mediated pyroptosis could aggravated blood-brain-barrier (BBB) damage after TBI. Moreover, Lee et al. (2019) showed evidence for inflammasome activation in microglia and infiltrating leukocytes after penetrating TBI, and the role of pyroptosis in the pathophysiology. However, the complex relationship among pyroptosis, ER stress and autophagy has not been elucidated.

Studies report that many growth factors, including bFGF, aFGF and EGF, improve the functional recovery by inhibiting ER stress or autophagy in spinal cord injury and TBI (Zhang et al., 2013a; Zhang et al., 2013b; Li et al., 2018; Xu et al., 2018; Wu et al., 2019). However, whether other growth factors also function *via* mediating ER stress and autophagy in the recovery of TBI remain unclear. As a neuroprotective factor, platelet derived growth factor (PDGF) plays a crucial role in neurological diseases (Padel et al., 2016; Cabezas et al., 2018; Osborne et al., 2018). Previous study have shown that PDGF decreased the number of TUNEL-positive neuron in hippocampal (Kawabe et al., 1997). Recently, Cabezas et al. demonstrated that PDGF mantained mitochondrial funtion and attenuated ROS production in astrocytes (Cabezas et al., 2018). In addition, it has been widely known that ER stress, autophgay and pyroptosis are closely associated with cellular homeostasis, mitochondrial homeostasis, cellular aging and inflammation condition. However, the role and mechanism of PDGF mediating the complex network among ER stress, autophagy and pyroptosis after TBI are still unclear.

In this study, we investigated the role of ER stress and autophagy at different time points of TBI, and further explored its underlying mechanism of PDGF on TBI-induced pyrotosis. During mechanism study, we found that ER stress and autophagy are involved in the neuroprotective of PDGF after TBI. Our current study suggests that PDGF holds a great promise to develop a new treatment for TBI recovery.

## Materials and methods

### Reagents and antibodies

Recombinant human PDGF was purchased from PeproTech (100-14B, United States). pPERK, ATF6, IRE1α, GRP78, PDI, VPS34, Beclin1, ATG5, LC3II, GAPDH and secondary antibodies were obtained from Abcam (Abcam, United States), PDI was purchased from Cell Signaling Technology (Danvers, MA, United States), NLRP3, Pro-Caspase1, Caspase1, GSDMD, GSDMDP30, IL-1 were obtained from Proteintech (Proteintech, China). Goat anti-rabbit and anti-mouse IgG-HRP were purchased from Cell Signaling Technology (Danvers, MA, United States). An enhanced chemiluminescence (ECL) kit was purchased from Bio-Rad (Hercules, CA, United States). H&E kit was purchased from Beyotime.

### Experimental animals and surgical procedures

A total of 90 C57BL/6 male mice (6–8 weeks old, weight = 18–25g, *n* = 3 in each group) were studied. All mice were supplied by the Animal Center of the Chinese Academy of Sciences (Shanghai, China). All experimental procedures were approved by the Ethics Committee of WenZhou Medical University and conformed to the Guide for the Care and Use of Laboratory Animals from the National Institutes of Health. To expose the cortex, a 5-mm diameter bone flap over the left parieto-temporal cortex was removed using a hand-held drill. TBI was induced by Impact One™ stereotaxic impactor (Leica, Milan, Italy). Each mouse was subjected to a controlled cortical impact using a 4-mm impact tip at a velocity of 4 m/s, a depth of 1 mm and a 150-ms impact duration. All experiments were carried out with minimal reduction in number of animals and their suffering.

### Drug administration

PDGF (PeproTech, United States) was used as drugs in this study. PDGF stock solution was diluted with 0.9% NaCl and administered *in situ* at a dose of 80 μg/kg after TBI. The mice were pre-administered with rapamycin (RAPA, 0.5 mg/kg, i. p.), tunicamycin (TM,10ug/kg,i.p.), 4-phenylbutyrate (4-PBA,100 mg/kg,i.p.), 3-methyladenine (3-MA,2.5 mg/kg, i. p.) for 3 days before injury.

### Cell culture and oxygen-glucose deprivation

HUVECs were expanded and maintained in Dulbecco’s modified Eagle’s medium (DMEM, Invitrogen, Carlsbad, CA, United States) supplemented with 10% Fetal Bovine Serum (FBS) (ScienCell, Carlsbad, CA, United States), and antibiotics (100 μg/ml streptomycin and 100 U/mL penicillin, ScienCell, Carlsbad, CA, United States). Cell were then incubated in a humidified atmosphere with 5% CO2 and 95% air at 37°C.For the OGD treatment, the cells were incubated with glucose-lower DMEM after treated with TM (3 µM) or Rapa (100 nM) and then placed in an anaerobic chamber which the levels of N2 was 95% for 6 h. The cells were pre-treated with PDGF (10 ng/ml) for 2 h before OGD. All experiments were performed in triplicate.

### Western blot

Protein from tissue and cell were homogenized in RIPA lysis buffer [1% Triton X-100, 1% deoxycholate, 0.1% SDS, 150 mM NaCl (pH 7.4) containing protease inhibitor cocktail (10 μL/ml; GE Healthcare Biosciences, Pittsburgh, PA, United States). The supernatants were collected after centrifugation at 12,000 rpm for 10 min at 4°C. The extracts were then quantified with BCA kit (Beyotime, China)**.** Following this, proteins were separated on a 10% or 12% gel and then were transferred into a polyvinylidene fluoride membrane (BioRad, Hercules, CA, United States). After blocking with 5% skim milk in TBST (Tris-buffered saline with 0.1% Tween-20) for 2 h at 37°C, the membranes were incubated with primary antibodies at 4°C overnight, and then incubated with horseradish peroxidase-conjugated secondary antibodies at room temperature for 1 h. Images were acquired with a Chemi DocXRS + Imaging System (Bio-Rad) and the bands were quantified by the Quantity-One software. All experiments were repeated for three times. The Western blot analysis of other cells was the same as the above procedure.

### Hematoxylin-eosin staining and brain water content

Transverse paraffin sections (5 mm thick) were mounted on poly-l-lysine-coated slides for histopathological examination using HE staining. Experimental steps were carried out according to manufactures’ instructions (C0105, Beyotime, China).

Mice were decapitated under deep anesthesia and perfusion at 21 days after TBI induction. The olfactory bulbs and brain stems were removed from their brains which were divided into right and left hemispheres. Each hemisphere was weighed immediately to determine the wet weight. The samples were dried at 72°C for 72 h to obtain dry weight. Brain water contents were calculated as follows: [(wet weight - dry weight)/wet weight] × 100%.

### Garcia neurobehavioral score

According to Garcia neurobehavioral score, the mice in each group were scored on day 1, day 7, day 14 and day 28 after TBI. The recovery of nerve function after TBI was observed by double blind method. Briefly, 1) spontaneous activity (0 ≤ 3 points). 2) symmetrical movement of limbs (0 ≤ 3 points). 3) forelimb extension (0 ≤ 3 points). 4) climbing (0–3 points). 5) body trunk reaction (0 ≤ 3 points). 6) tentacles reaction (0 ≤ 3 points). 7) lateral rotation reaction (0 ≤ 3 points).

### Immunofluorescence staining

The tissues or cells were fixed in 4% paraformaldehyde (PFA) for 24 h or 30 min. The brain tissues embedded in paraffin, and then cut into 5 µm sections. The sections were deparaffinized, rehydrated. The brain tissues and cells were incubated with 5% BSA at 37°C for 30 min and then were incubated overnight at 4°C with the primary antibodies. The tissues and cells were washed three times with PBST and incubated with Alexa Fluor 488 donkey anti-rabbit secondary antibodies for 1 h at 37°C in the next day. Nuclei were stained with DAPI for 10 min. All images were captured under fluorescence microscope (Nikon, A1PLUS).

### Statistical analysis

The data were presented as mean ± standard Deviation (SD). Statistical significance was performed using one-way analysis of variance (ANOVA) and Dunnett’s post hoc test when make comparison among three or more groups. *p* values < 0.05 were considered as statistically significant.

## Results

### Activation of endoplasmic reticulum stress and autophagy on traumatic brain injury

**FIGURE 1:**
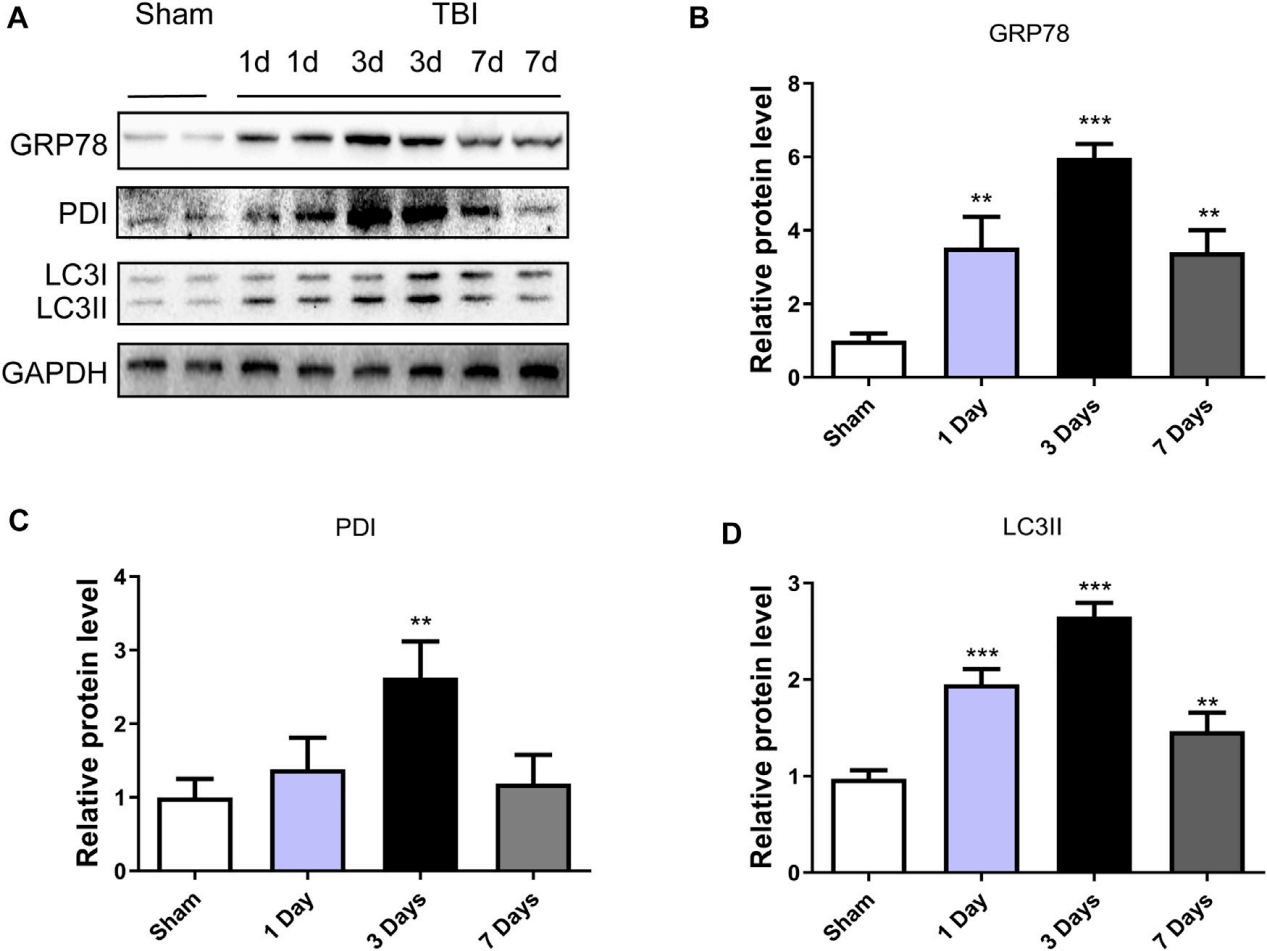
TBI activates ER stress and autophagy. **(A)** Representative western blot analyses of GRP78, PDI and LC3I/II in the cerebral cortex from sham group and 1 day, 3 days and 7 days post-injury group. **(B–D)** Quantification of GRP78, PDI and LC3II in the ipsilateral brain cortex at 1d, 3d and 7d after TBI. Data represent the mean ± SD, *n* = 3, **p < 0.01 and ***p < 0.001 vs. the sham group.

To explore whether ER stress and autophagy were involved in TBI, the expression levels of ER stress markers (GRP78 and PDI) and autophagy markers (LC3II) in a mice model of TBI were detected at different time points. Figures 1A–C showed that the GRP78 and PDI expressions were significantly increased after TBI and with 3d post-surgery had the most prominent upregulation (*p* < 0.001, *p* < 0.01, respectively). The same results were observed in the LC3II/I (*p* < 0.001) expressions. These results suggested that ER stress and autophagy were activated after TBI, and three dpi mice model was chosen for the subsequent experiments.

### The activation of traumatic brain injury-induced endoplasmic reticulum stress and autophagy after platelet derived growth factor treatment

**FIGURE 2:**
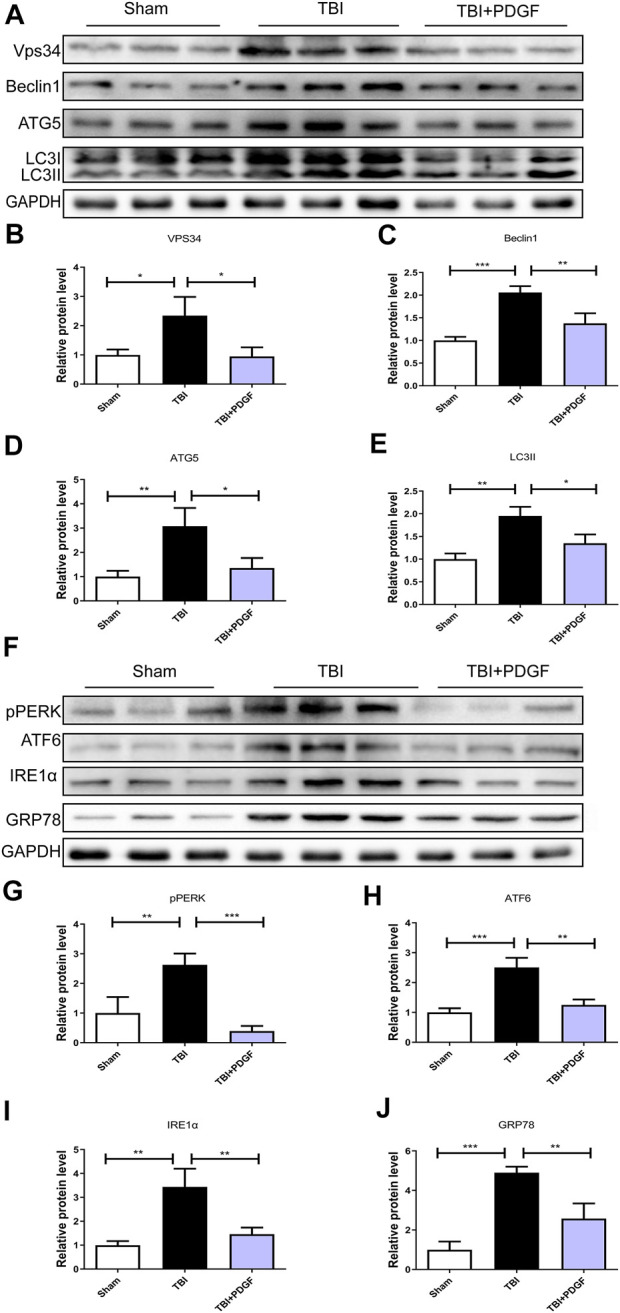
PDGF treatment significantly attenuates TBI-induced ER stress and autophagy. **(A)** Representative western blot analyses of VPS34, Beclin1, ATG5, and LC3I/II in the cerebral cortex at 3d after TBI. **(B–E)** Quantification of VPS34, Beclin1, ATG5, LC3II in the ipsilateral brain cortex at 3 days after TBI. Data represent the mean ± SD, *n* = 3, *p < 0.05, **p < 0.01 and ***p < 0.001 vs. the indicated group. **(F)** Representative western blot analyses of pPERK, ATF6, IRE1α and GRP78 in the cerebral cortex at 3 days after TBI. **(G–J)** Quantification of pPERK, ATF6, IRE1α and GRP78 in the ipsilateral brain cortex at 3 days after TBI. Data represent the mean ± SD, *n* = 3, **p < 0.01 and ***p < 0.001 vs. the indicated group.

This study was designed to evaluate the neuroprotective effect of PDGF on ER stress and autophagy in TBI. As shown in Figures 2A–E, the expressions of autophagy-related proteins like VPS34 (*p* < 0.05), Beclin1(*p* < 0.001), ATG5(*p* < 0.01) and LC3II (*p* < 0.01) were significant increased after TBI when compared with the sham group. While compared with the TBI group, the expressions of proteins were obviously decreased upon PDGF treatment (*p* < 0.05 for VPS34, *p* < 0.01 for Beclin1, *p* < 0.05 for ATG5, *p* < 0.05 for LC3II). The expression levels of ER stress-related proteins were further detected. PDGF treatment significantly blocked TBI-induced increase of pERK (*p* < 0.001), ATF6 (*p* < 0.01), IREα (*p* < 0.01) and GRP78 (*p* < 0.01) expressions **(** Figures 2F–J **)**. These results suggested that the PDGF treatment significantly inhibited the autophagy and ER stress after TBI.

### Pyroptosis is associated with traumatic brain injury-induced endoplasmic reticulum stress and autophagy after platelet derived growth factor treatment

**FIGURE 3:**
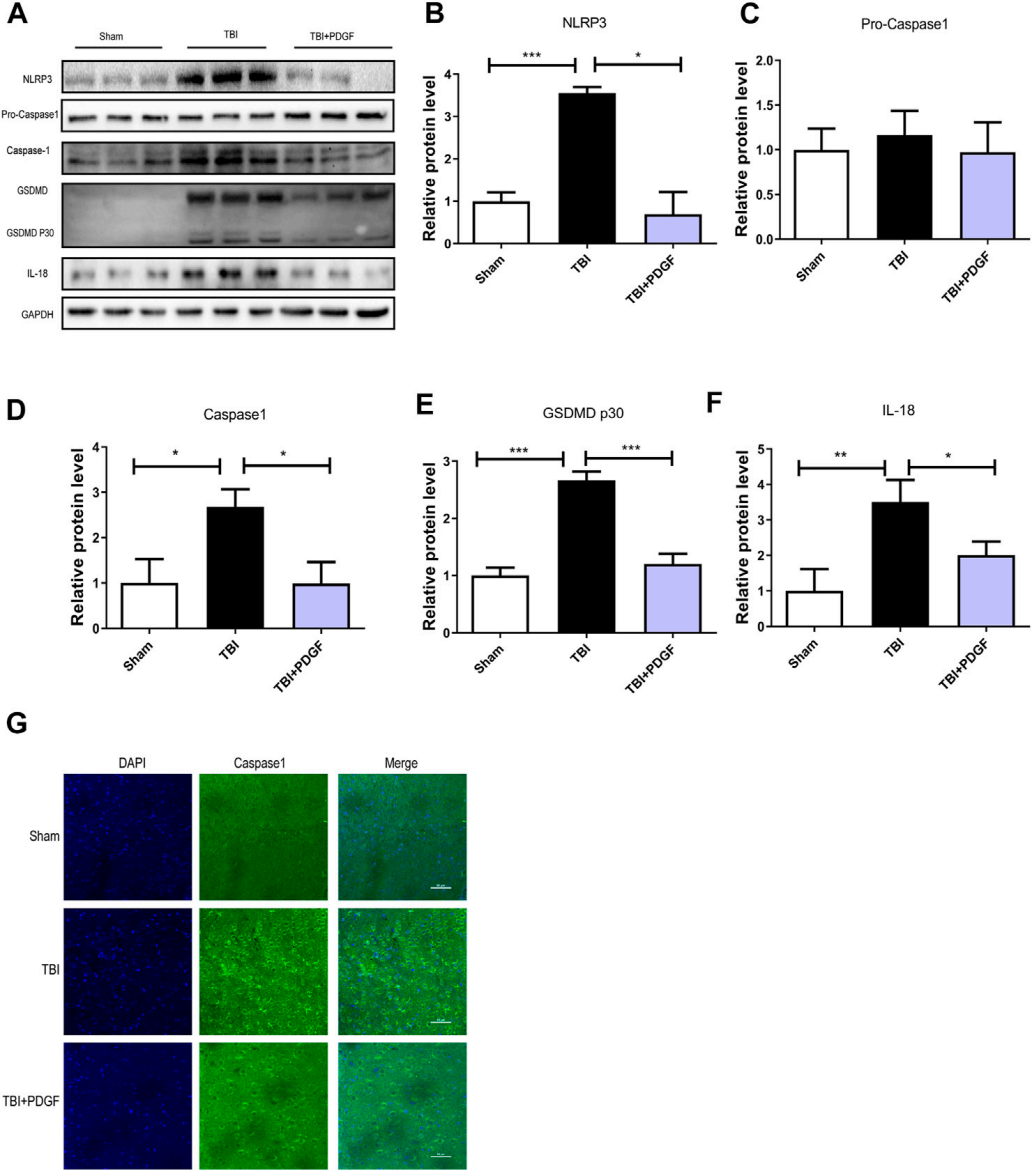
PDGF treatment reverses Pyroptosis after TBI. **(A)** Representative western blot analyses of NLRP3, Pro-caspase1, Caspase1, GSDMD, GSDMDP30 and IL-18 in the cerebral cortex at 3d after TBI. **(B–F)** Quantification of NLRP3, Pro-caspase1, Caspase1, GSDMDP30 and IL-18 in the ipsilateral brain cortex at 3d after TBI. Data represent the mean ± SD, *n* = 3, *p < 0.05, **p < 0.01 and ***p < 0.001 vs. the indicated group. **(G)**Representation and quantification of immunofluorescence staining of Caspase1 (green) in the cortex at 3 days post-injury.

**FIGURE 4:**
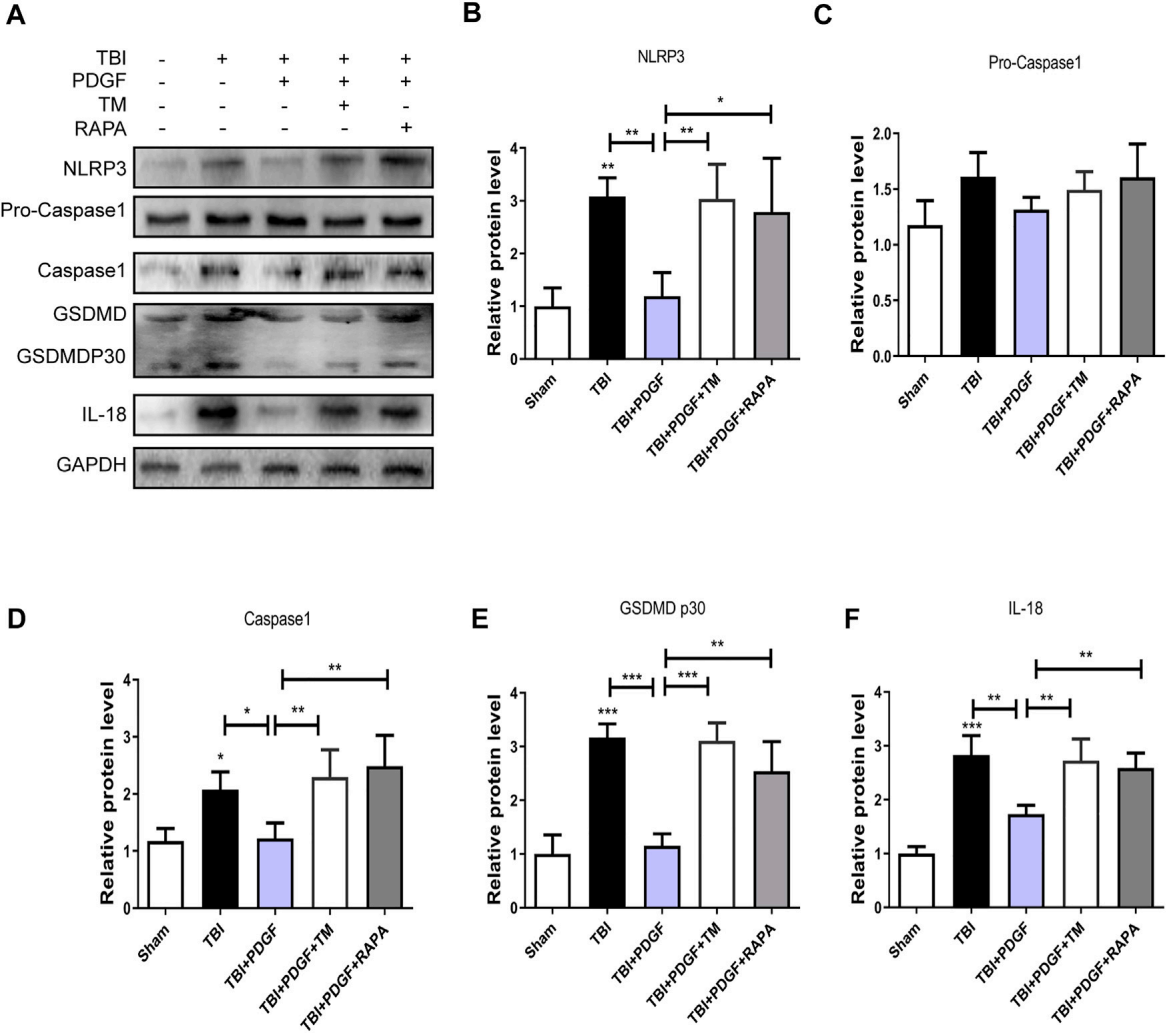
PDGF treatment inhibits the pyroptosis by blocking the ER stress and autophagy after TBI. **(A)** Representative western blot analyses of NLRP3, Pro-caspase1, Caspase1, GSDMD, GSDMDP30 and IL-18 in the cerebral cortex at 3 days after TBI. **(B–F)** Quantification of NLRP3, Pro-caspase1, Caspase1, GSDMDP30, and IL-18 in the ipsilateral brain cortex at 3 days after TBI. Data represent the mean ± SD, *n* = 3, *p < 0.05, **p < 0.01 and ***p < 0.001 vs. the indicated group.

To determine whether PDGF inhibited pyroptosis after TBI, we examined the expressions of NLRP3, Pro-caspase1, Caspase1, GSDMD, GSDMDP30, and IL-18. Figures 3A–F showed that the expressions of the pyroptosis-related proteins were significantly increased after TBI (*p* < 0.001 for NLRP3, *p* < 0.05 for Caspase1, *p* < 0.001 for GSDMP30, *p* < 0.01 for IL-18), and remarkably blocked by PDGF treatment (*p* < 0.05 for NLRP3, *p* < 0.05 for Caspase1, *p* < 0.001 for GSDMP30, *p* < 0.05 for IL-18). Next, we used immunofluorescence to detect Caspase-1 in mice. Results showed that the number of Caspase-1 positive in the PDGF treatment was decreased compared with the TBI group. The results were further validated by using TM and RAPA. As is shown in Figures 4A–F **,** the expression of pyroptosis-related proteins were significantly increased after co-treated with TM or RAPA treatment than in the PDGF group (*p* < 0.01 in the TBI + PDGF + TM group, *p* < 0.05 in the TBI + PDGF + RAPA group for NLRP3; *p* < 0.01 both in the TBI + PDGF + TM group and TBI + PDGF + RAPA group for Caspase1; *p* < 0.001 in the TBI + PDGF + TM group, *p* < 0.01 in the TBI + PDGF + RAPA group for GSDMP30; *p* < 0.01 both in the TBI + PDGF + TM group and TBI + PDGF + RAPA group for IL-18). The above results demonstrated that the PDGF treatment inhibited pyroptosis by inhibiting ER stress and autophagy.

### The endoplasmic reticulum stress regulates the autophagy in traumatic brain injury

**FIGURE 5:**
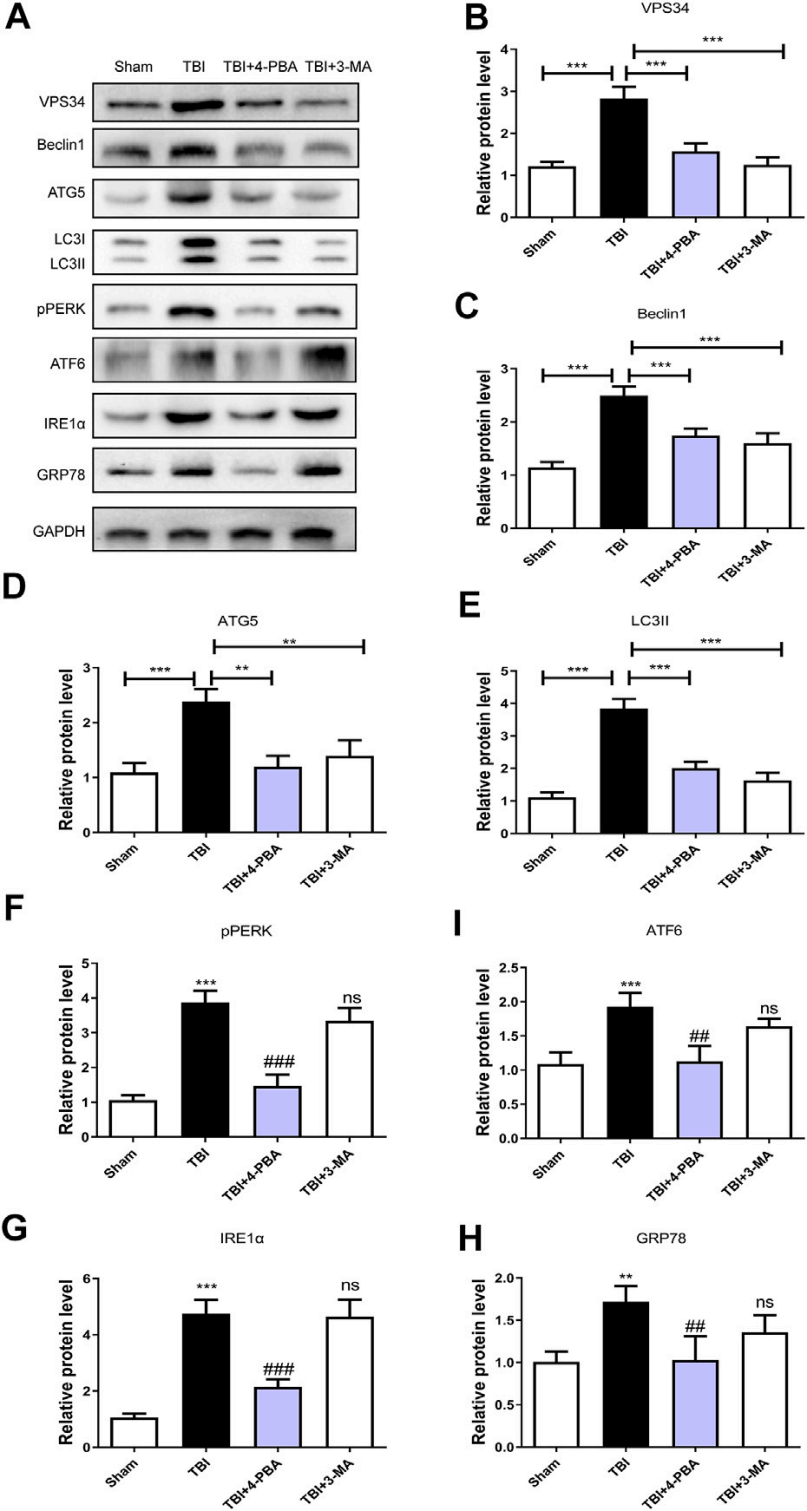
The ER stress regulates the autophagy in TBI. **(A)** Representative western blot analyses of VPS34, Beclin1, ATG5, LC3I/II, pPERK, ATF6, IRE1α, and GRP78 in the cerebral cortex at 3 days after TBI. **(B–H)** Quantification of VPS34, Beclin1, ATG5, LC3II, pPERK, ATF6, IRE1α and GRP78 in the ipsilateral brain cortex at 3 days after TBI. Data represent the mean ± SD, *n* = 3, **p < 0.01 and ***p < 0.001 vs. the indicated group; **p < 0.01 and ***p < 0.001 vs. the sham group. ^##^ *p* < 0.01, ###*p* < 0.001 and ns vs. the TBI group.

To further investigate the relationship between ER stress and autophagy during TBI, 4-PBA (a classical ER stress inhibitor) and 3-MA (a classical autophagy inhibitor) were used to treatment for TBI. As shown in Figures 5A–H, 4-PBA could inhibit the ER stress and autophagy through the reduction of VPS34 (*p* < 0.001), Beclin1 (*p* < 0.001), ATG5 (*p* < 0.01), LC3II (*p* < 0.001), pERK (*p* < 0.001), ATF6 (*p* < 0.01), IREα (*p* < 0.001) and GRP78 (*p* < 0.01). However, the 3-MA only inhibited the autophagy (*p* < 0.001 for VPS34; *p* < 0.001 for Beclin1; *p* < 0.01 for ATG5; *p* < 0.001 for LC3II). These results suggested that TBI-induced ER stress significantly triggered autophagy.

### Platelet derived growth factor treatment improves the functional recovery through inhibiting endoplasmic reticulum stress and autophagy after traumatic brain injury

**FIGURE 6:**
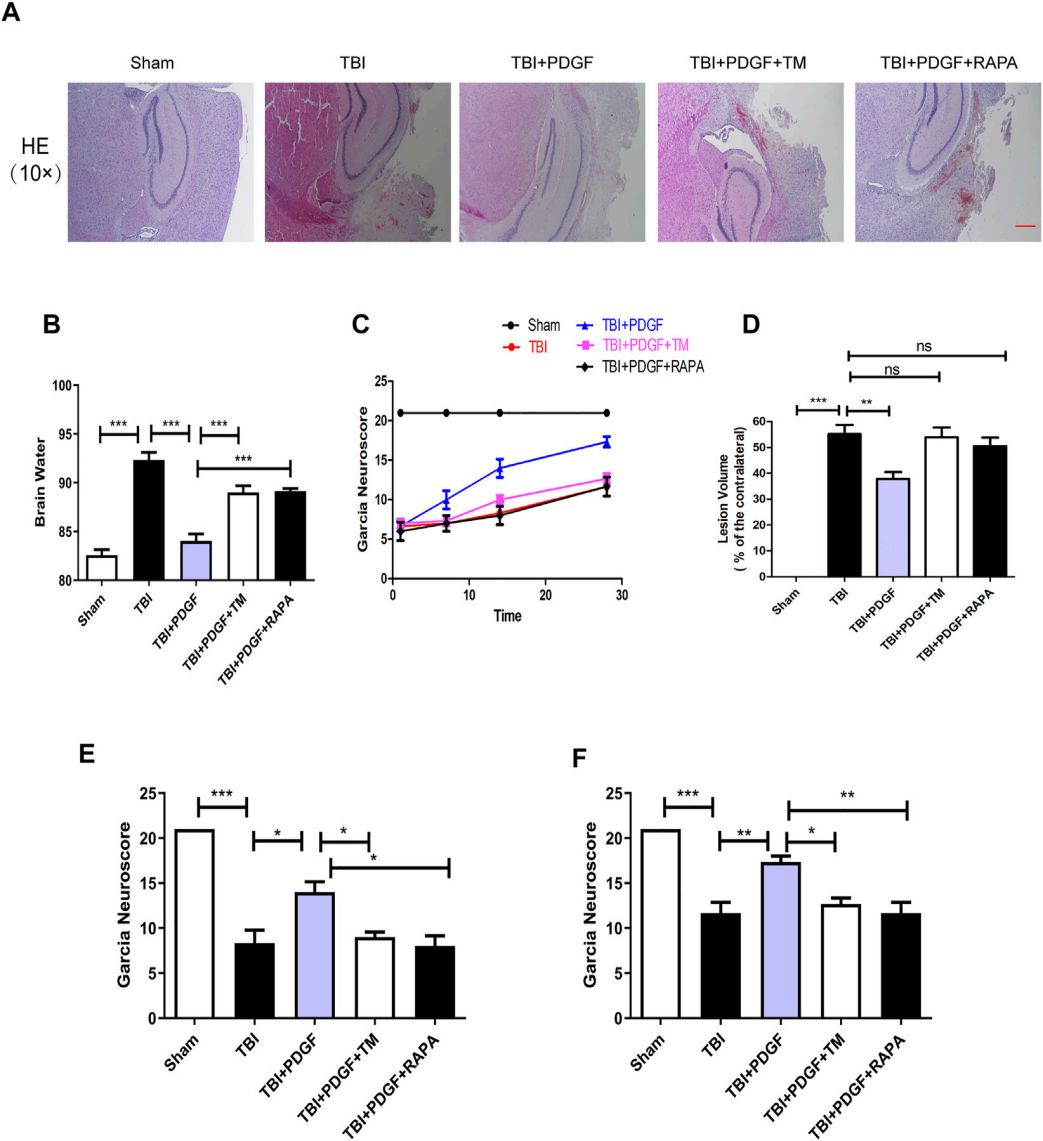
The PDGF treatment improves the functional recovery through ER stress and autophagy after TBI. **(A)** Representative images of hematoxylin and eosin **(H&E)** staining in the cortex at 21d post-TBI. Scar bar = 100 μm. **(B)** Quantification of brain water content in the ipsilateral brain cortex at 3d after TBI. **(C)** Garcia test evaluation at 1 day, 7 days, 14 days, and 28 days after TBI. **(D) **Quantification of lesion volume after H&E staining at 21 days post-TBI; **(E) **Garcia test evaluation at 14 days after TBI. **(F)** Garcia test evaluation at 28 days after TBI. Data represent the mean ± SD, *n* = 3, *p < 0.05, **p < 0.01 and ***p < 0.001 vs. the indicated group.

To evaluate the therapeutic role of PDGF in the treatment of TBI, PDGF, TM, and RAPA were administered immediately following TBI. H&E staining was performed to assess the histological morphology in each group. Figure 6D showed that severe cerebral cortex tissue loss in the TBI group was more obvious than the sham group (*p* < 0.001), and the PDGF-treated groups showed less tissue damage (*p* < 0.01) while TM and RAPA offset the effect of PDGF. Brain water content was detected at 3 days after TBI. As shown in Figure 6B, the brain water content of the mice in TBI group was obviously increased compared to the sham group (*p* < 0.001). Consistent with the results of H&E, the brain water contents of the PDGF-treated TBI groups were reduced relative to the TBI group (*p* < 0.001) while PDGF plus TM and PDGF plus RAPA also offset the effect of PDGF. Motor function recovery was estimated for 28 days after injury using the 21-point Garcia test. As shown in Figure 6C, the sham group had an average score of 21, representing a normal motor function. The Garcia scores were not significantly different between the treated and TBI group at days 1 and 7. Interestingly, there was significantly increased in the PDGF-treated group compared with those in TBI (*p* < 0.05 in days 14; *p* < 0.01 in days 28) during 21-point Garcia test. However, the Garcia scores in the TBI + PDGF + TM significantly decreased when compared with the TBI + PDGF group (*p* < 0.05 both in days 14 and day 28). Meanwhile, the Garcia scores in TBI + PDGF + RAPA group was consisted with the TBI + PDGF + TM group (*p* < 0.05 in days 14, *p* < 0.01 in days 28, compared with the PDGF group). These results suggested PDGF improves the functional recovery by inhibiting ER stress and autophagy after TBI.

### Platelet derived growth factor administration inhibits pyroptosis by inhibiting the endoplasmic reticulum stress and autophagy in OGD-Treated HBMECs

**FIGURE 7:**
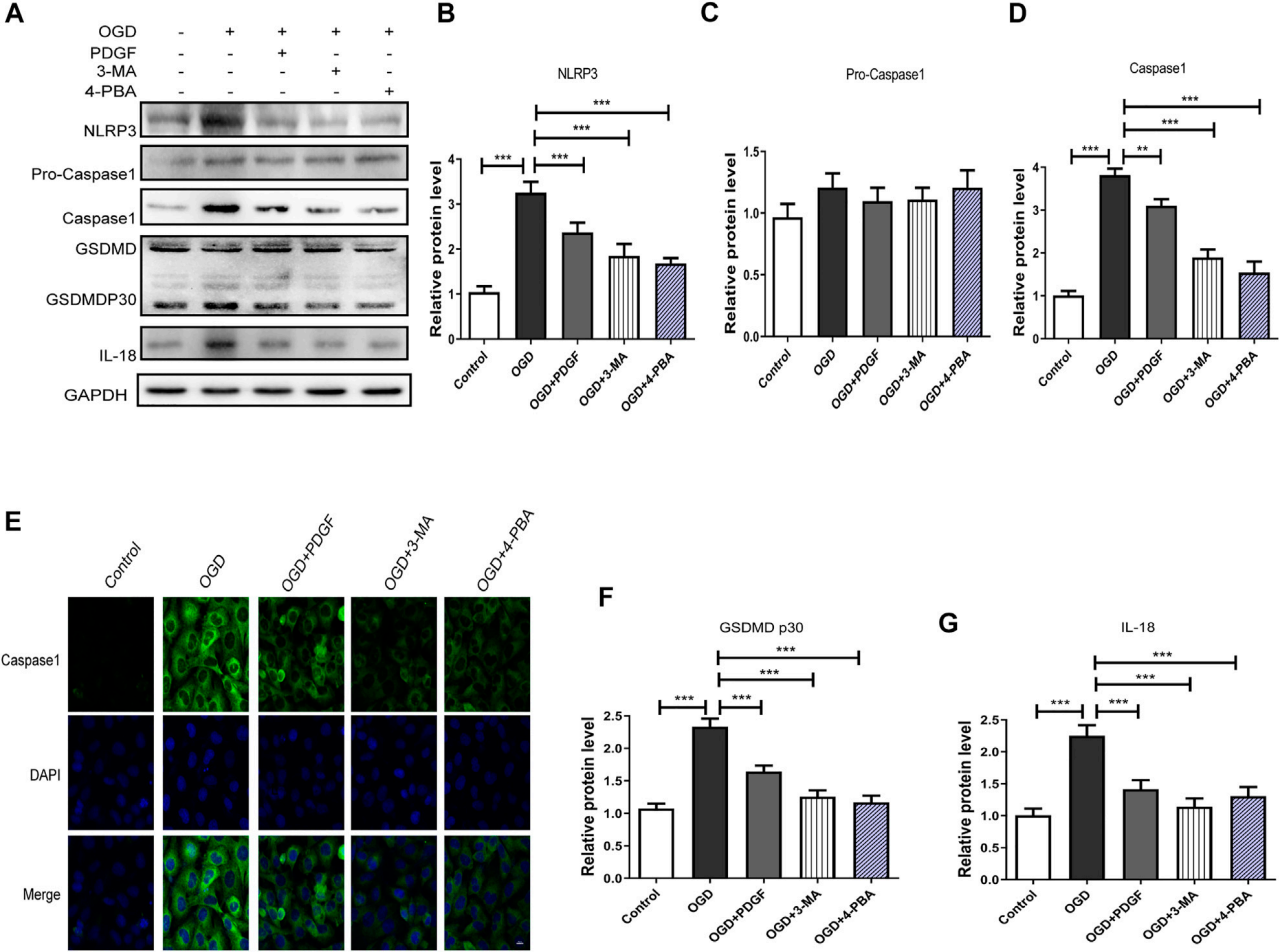
PDGF administration inhibits pyroptosis in OGD-treated HBMECs. **(A)** Representative western blot analyses of NLRP3, Pro-caspase1, caspase1, GSDMD, GSDMDP30, IL-18 in HBMECs after OGD. **(B–D)** Quantification of NLRP3, Pro-caspase1 and caspase1 in the HBMECs after OGD. Data represent the mean ± SD, *n* = 3 **p < 0.01 and ***P<0.001 vs. the indicated group; **(E)** Representation and quantification of immunofluorescence staining of Caspase1 (green) in the cortex at 3 days post-injury. **(F–G)** Quantification of GSDMDP30 and IL-18 in the HBMECs after OGD. Data represent the mean ± SD, *n* = 3 ***p < 0.001 vs. the indicated group.

We next used 3-MA and 4-PBA to explore the mechanism of PDGF therapeutic role in HBMECs after OGD. As shown in Figures 7A–G, the pyroptosis-related proteins expression were significantly decreased in PDGF-treated group compared to that of the OGD group (*p* < 0.001 for NLRP3; *p* < 0.01 for Caspase1; *p* < 0.001 for GDSMDP30; *p* < 0.001 for IL-18), which were similar to the effect of 3-MA and 4-PBA. Then we used immunofluorescence to detect Caspase1 *in vitro*. The results showed that the intensity of Caspase1-positive in the OGD group was increased relative to the PDGF group, which was consistent with the western bolt results. In conclusion, these results suggested that PDGF was able to inhibits pyroptosis by down-regulating the level of ER stress and autophagy in OGD mode.

## Discussion

TBI is a leading cause of morbidity in modern society. Numerous studies have elucidated the pathophysiological mechanisms of this fatal disease in the past decades (Mattson and Scheff, 1994). It is generally accepted that the secondary insults after TBI, such as excitotoxicity, oxidative stress, inflammation and apoptosis, aggravate the primary injury which occurs during impact (Xiong et al., 2018). Therefore, it is important to explore a new therapeutic drug for TBI to reduce secondary damage.

It has been evidenced that the PDGF has neuroprotective effects on nervous diseases *via* anti-oxidative stress, protecting mitochondria and angiogenesis, and so on (Zheng et al., 2010; Cabezas et al., 2018). PDGF exerts diverse functions in the nervous system, covering neurogenesis, cell survival, synaptogenesis, modulation of ligand-gated ion channels, and development of specific types of neurons (Funa and Sasahara, 2014; Sil et al., 2018). This study was designed to evaluate the neuroprotective effect of PDGF on TBI. Our results indicated that PDGF could improve functional recovery after TBI. Further studies demonstrated that PDGF functioned *via* inhibiting pyroptosis. Pyroptosis is a highly specific type of inflammatory programmed cell death, which is different from necrosis or apoptosis. Liu et al. has reported that lack of caspase-1 leads to decreased inflammatory response in the injured cortex, and the inhibition of pyroptosis alleviates neuroinflammation and associated neurological deficits in the acute phase of TBI (Liu et al., 2018). In addition, Lee et al. (2019) has also reported that the inflammasome activation in microglia and leukocyte infiltration after penetrating TBI and a role for pyroptotic cell death in the pathophysiology. Consistent with previous studies, our study had also substantiated pyroptosis in TBI, and PDGF could decrease the expression of pyroptosis-related protein, such as NLRP3,Caspase1, GSDMDP30, and IL-18. However, it remains not fully understood the underlying molecular mechanism of PDGF regulating pyroptosis.

The ER is an intracellular organelle that functions in the biosynthesis of membranes, secretory proteins, lipids and sterols, and the maintenance of intracellular calcium homeostasis (Shen et al., 2004). Numerous studies have demonstrated that ER stress is involved in many diseases including neurodegenerative disorders, metabolic diseases, and inflammation (Lindholm et al., 2006). Recent studies indicated that attenuation of ER stress could inhibit pyroptosis (Lebeaupin et al., 2015). Therfore, we evaluated the role of ER stress in the effect of PDGF on pyroptosis, and found that PDGF treatment inhibited the activation of ER stress. PDGF treatment alleviated the activation of pPERK, ATF6, IRE1α, GRP78. Furthermore, the activation of ER stress with TM significantly reversed the inhibition of PDGF on pyroptosis and motor functional recovery after TBI. These data revealed that the PDGF treatment alleviated pyroptosis through regualting the ER stress.

Previous studies paid more attention to the role of autophagy after TBI, and revealed that the inhibition of autophagy could improve the functional recovery (Xu et al., 2018; Wu et al., 2019). Zarogoulidis et al.reported that autophagy induced apoptosis *via* enhancing tumor necrosis factor-related apoptosis-inducing ligand (TRAIL) (Zarogoulidis et al., 2016). Wang J et al. reported that PDGF induces autophagy through the Beclin-1 pathway to regulate the biological behavior of oral mucosal fibroblasts (Wang J. et al., 2021). Nevertheless, the relationship between autophagy and PDGF in TBI requires further study. Therefore, we examined the effect of PDGF on autophagy-mediated pyroptosis signaling pathways after TBI, and the results were similar to the PDGF on ER stress-mediated pyroptosis. Prior studies have demonstrated that there are complex interaction between autophagy and ER stress in the neurodegenerative disease (Esmaeili et al., 2022). Numerous studies reported that ER stress triggers autophagy after SCI (Zhou et al., 2017; Wang et al., 2018; Bisicchia et al., 2022). However, whether ER stress mediates autophagy during PDGF treatment for TBI remains unclear. Some studies have indicated that activated ER stress could trigger autophagy (Ogata et al., 2006; Zhou et al., 2017). We found that the 4-PBA could inhibit the ER stress and autophagy after TBI, while 3-MA only inhibited the autophagy. These results implied that PDGF treatment blocked ER stress-mediated pyroptosis and autophagy, hence ameliorating TBI.

In summary, our study proved that PDGF significantly inhibits pyroptosis, thus improves functional recovery after TBI. The ER stress regulates the autophagy which is involved in the PDGF treatment for TBI. Overall, these findings indicated that PDGF inhibits pyroptosis *via* inhibition of ER stress-mediated autophagy in the TBI.

## Conclusion

This study demonstrated that PDGF promotes the recovery of TBI, and found that the PDGF could inhibit pyroptosis after TBI. In addition, we also investigated the possible mechanisms involved in PDGF treatment. Furthermore, this study revealed that its mechanisms are possibly through inhibiting autophagy and endoplasmic reticulum stress. In conclusion, our study suggests that PDGF promotes the recovery of TBI by inhibiting pyroptosis.

## Data Availability

The original contributions presented in the study are included in the article/supplementary material, further inquiries can be directed to the corresponding authors.

## References

[B1] Annadurai T. Sil S. Tripathi A. Chivero Ernest T. Palsamy. P. Buch S. (2020). Targeting endoplasmic reticulum stress and autophagy as therapeutic approaches for neurological diseases. Int. Rev. Cell Mol. Biol. 350, 285–325. 10.1016/bs.ircmb.2019.11.001 32138902

[B2] Arruri V. Raghu V. (2022). Role of autophagy and transcriptome regulation in acute brain injury. Exp. Neurol. 352, 114032. 10.1016/j.expneurol.2022.114032 35259350 PMC9187300

[B3] Bisicchia E. Mastrantonio R. Nobili A. Palazzo C. La Barbera L. Latini L. (2022). Restoration of ER proteostasis attenuates remote apoptotic cell death after spinal cord injury by reducing autophagosome overload. Cell Death Dis. 13 (4), 381. 10.1038/s41419-022-04830-9 35444186 PMC9021197

[B4] Butterfield D. A. Reed T. T. (2016). Lipid peroxidation and tyrosine nitration in traumatic brain injury: Insights into secondary injury from redox proteomics. Proteomics. Clin. Appl. 10 (12), 1191–1204. 10.1002/prca.201600003 27588567

[B5] Cabezas R. Vega-Vela N. E. Gonzalez-Sanmiguel J. Gonzalez J. Esquinas P. Echeverria V. (2018). PDGF-BB preserves mitochondrial morphology, attenuates ROS production, and upregulates neuroglobin in an astrocytic model under rotenone insult. Mol. Neurobiol. 55 (4), 3085–3095. 10.1007/s12035-017-0567-6 28466269

[B6] Esmaeili Y. Yarjanli Z. Pakniya F. Bidram E. Los M. J. Eshraghi M. (2022). Targeting autophagy, oxidative stress, and ER stress for neurodegenerative disease treatment. J. Control. Release 345, 147–175. 10.1016/j.jconrel.2022.03.001 35248646

[B7] Funa K. Sasahara M. (2014). The roles of PDGF in development and during neurogenesis in the normal and diseased nervous system. J. Neuroimmune Pharmacol. 9 (2), 168–181. 10.1007/s11481-013-9479-z 23771592 PMC3955130

[B8] Gao Y. Zhang M. Y. Wang T. Fan Y. Y. Yu L. S. Ye G. H. (2018). IL-33/ST2L signaling provides neuroprotection through inhibiting autophagy, endoplasmic reticulum stress, and apoptosis in a mouse model of traumatic brain injury. Front. Cell. Neurosci. 12, 95. 10.3389/fncel.2018.00095 29922130 PMC5996884

[B9] Garvin R. Mangat H. S. (2017). Emergency neurological life support: Severe traumatic brain injury. Neurocrit. Care 27 (1), 159–169. 10.1007/s12028-017-0461-0 28913754

[B10] Ge X. Li W. Huang S. Yin Z. Xu X. Chen F. (2018). The pathological role of NLRs and AIM2 inflammasome-mediated pyroptosis in damaged blood-brain barrier after traumatic brain injury. Brain Res. 1697, 10–20. 10.1016/j.brainres.2018.06.008 29886252

[B11] Hughes D. Mallucci G. R. (2019). The unfolded protein response in neurodegenerative disorders - therapeutic modulation of the PERK pathway. FEBS J. 286 (2), 342–355. 10.1111/febs.14422 29476642

[B12] Kawabe T. Wen T. C. Matsuda S. Ishihara K. Otsuda H. Sakanaka M. (1997). Platelet-derived growth factor prevents ischemia-induced neuronal injuries *in vivo* . Neurosci. Res. 29 (4), 335–343. 10.1016/s0168-0102(97)00105-3 9527625

[B13] Lebeaupin C. Proics E. De Bieville C. H. Rousseau D. Bonnafous S. Patouraux S. (2015). ER stress induces NLRP3 inflammasome activation and hepatocyte death. Cell Death Dis. 6, e1879. 10.1038/cddis.2015.248 26355342 PMC4650444

[B14] Lee S. W. De Rivero Vaccari J. P. Truettner J. S. Dietrich W. D. Keane R. W. (2019). The role of microglial inflammasome activation in pyroptotic cell death following penetrating traumatic brain injury. J. Neuroinflammation 16 (1), 27. 10.1186/s12974-019-1423-6 30736791 PMC6367831

[B15] Li J. Wang Q. Cai H. He Z. Wang H. Chen J. (2018). FGF1 improves functional recovery through inducing PRDX1 to regulate autophagy and anti-ROS after spinal cord injury. J. Cell. Mol. Med. 22 (5), 2727–2738. 10.1111/jcmm.13566 29512938 PMC5908106

[B16] Lindholm D. Wootz H. Korhonen L. (2006). ER stress and neurodegenerative diseases. Cell Death Differ. 13 (3), 385–392. 10.1038/sj.cdd.4401778 16397584

[B17] Liu W. Chen Y. Meng J. Wu M. Bi F. Chang C. (2018). Ablation of caspase-1 protects against TBI-induced pyroptosis *in vitro* and *in vivo* . J. Neuroinflammation 15 (1), 48. 10.1186/s12974-018-1083-y 29458437 PMC5817788

[B18] Mattson M. P. Scheff S. W. (1994). Endogenous neuroprotection factors and traumatic brain injury: Mechanisms of action and implications for therapy. J. Neurotrauma 11 (1), 3–33. 10.1089/neu.1994.11.3 8201625

[B19] Ogata M. Hino S. Saito A. Morikawa K. Kondo S. Kanemoto S. (2006). Autophagy is activated for cell survival after endoplasmic reticulum stress. Mol. Cell. Biol. 26 (24), 9220–9231. 10.1128/MCB.01453-06 17030611 PMC1698520

[B20] Osborne A. Sanderson J. Martin K. R. (2018). Neuroprotective effects of human mesenchymal stem cells and platelet-derived growth factor on human retinal ganglion cells. Stem Cells 36 (1), 65–78. 10.1002/stem.2722 29044808 PMC5765520

[B21] Padel T. Ozen I. Boix J. Barbariga M. Gaceb A. Roth M. (2016). Platelet-derived growth factor-BB has neurorestorative effects and modulates the pericyte response in a partial 6-hydroxydopamine lesion mouse model of Parkinson's disease. Neurobiol. Dis. 94, 95–105. 10.1016/j.nbd.2016.06.002 27288154

[B22] Shen X. Zhang K. Kaufman R. J. (2004). The unfolded protein response-a stress signaling pathway of the endoplasmic reticulum. J. Chem. Neuroanat. 28 (1-2), 79–92. 10.1016/j.jchemneu.2004.02.006 15363493

[B23] Sil S. Periyasamy P. Thangaraj A. Chivero E. T. Buch S. (2018). PDGF/PDGFR axis in the neural systems. Mol. Asp. Med. 62, 63–74. 10.1016/j.mam.2018.01.006 PMC6003857 29409855

[B24] Srivastava A. K. Cox C. S. (2018). Pre-clinical and clinical methods in brain trauma research. New York, USA: Springer New York.

[B25] Sun G. Z. Gao F. F. Zhao Z. M. Sun H. Xu W. Wu L. W. (2016). Endoplasmic reticulum stress-induced apoptosis in the penumbra aggravates secondary damage in rats with traumatic brain injury. Neural Regen. Res. 11 (8), 1260–1266. 10.4103/1673-5374.189190 27651773 PMC5020824

[B26] Wang D. Y. Hong M. Y. Pei J. Gao Y. H. Zheng Y. Xu X. (2021a). ER stress mediated-autophagy contributes to neurological dysfunction in traumatic brain injury via the ATF6 UPR signaling pathway. Mol. Med. Rep. 23 (4), 247. 10.3892/mmr.2021.11886 33537827

[B27] Wang H. Wu Y. Han W. Li J. Xu K. Li Z. (2018). Hydrogen sulfide Ameliorates blood-spinal cord barrier disruption and improves functional recovery by inhibiting endoplasmic reticulum stress-dependent autophagy. Front. Pharmacol. 9, 858. 10.3389/fphar.2018.00858 30210332 PMC6121111

[B28] Wang J. Yang L. You J. Wen D. Yang B. Jiang C. (2021b). Platelet-derived growth factor regulates the biological behavior of oral mucosal fibroblasts by inducing cell autophagy and its mechanism. J. Inflamm. Res. 14, 3405–3417. 10.2147/JIR.S313910 34305405 PMC8297405

[B29] Wang Z. F. Gao C. Chen W. Gao Y. Wang H. C. Meng Y. (2019). Salubrinal offers neuroprotection through suppressing endoplasmic reticulum stress, autophagy and apoptosis in a mouse traumatic brain injury model. Neurobiol. Learn. Mem. 161, 12–25. 10.1016/j.nlm.2019.03.002 30851432

[B30] Wu F. Xu K. Liu L. Zhang K. Xia L. Zhang M. (2019). Vitamin B12 enhances nerve repair and improves functional recovery after traumatic brain injury by inhibiting ER stress-induced neuron injury. Front. Pharmacol. 10, 406. 10.3389/fphar.2019.00406 31105562 PMC6491933

[B31] Xiong Y. Mahmood A. Chopp M. (2018). Current understanding of neuroinflammation after traumatic brain injury and cell-based therapeutic opportunities. Chin. J. Traumatol. 21 (3), 137–151. 10.1016/j.cjtee.2018.02.003 29764704 PMC6034172

[B32] Xu K. Wu F. Xu K. Li Z. Wei X. Lu Q. (2018). NaHS restores mitochondrial function and inhibits autophagy by activating the PI3K/Akt/mTOR signalling pathway to improve functional recovery after traumatic brain injury. Chem. Biol. Interact. 286, 96–105. 10.1016/j.cbi.2018.02.028 29567101

[B33] Yin Y. Sun G. Li E. Kiselyov K. Sun D. (2017). ER stress and impaired autophagy flux in neuronal degeneration and brain injury. Ageing Res. Rev. 34, 3–14. 10.1016/j.arr.2016.08.008 27594375 PMC5250579

[B34] Zarogoulidis P. Petanidis S. Domvri K. Kioseoglou E. Anestakis D. Freitag L. (2016). Autophagy inhibition upregulates CD4(+) tumor infiltrating lymphocyte expression via miR-155 regulation and TRAIL activation. Mol. Oncol. 10 (10), 1516–1531. 10.1016/j.molonc.2016.08.005 27692344 PMC5423126

[B35] Zeiler F. A. Mcfadyen C. Newcombe V. F. J. Synnot A. Donoghue E. L. Ripatti S. (2021). Genetic influences on patient-oriented outcomes in traumatic brain injury: A living systematic review of non-apolipoprotein E single-nucleotide polymorphisms. J. Neurotrauma 38 (8), 1107–1123. 10.1089/neu.2017.5583 29799308 PMC8054522

[B36] Zhang H. Y. Wang Z. G. Wu F. Z. Kong X. X. Yang J. Lin B. B. (2013a). Regulation of autophagy and ubiquitinated protein accumulation by bFGF promotes functional recovery and neural protection in a rat model of spinal cord injury. Mol. Neurobiol. 48 (3), 452–464. 10.1007/s12035-013-8432-8 23516099

[B37] Zhang H. Y. Zhang X. Wang Z. G. Shi H. X. Wu F. Z. Lin B. B. (2013b). Exogenous basic fibroblast growth factor inhibits ER stress-induced apoptosis and improves recovery from spinal cord injury. CNS Neurosci. Ther. 19 (1), 20–29. 10.1111/cns.12013 23082997 PMC6493620

[B38] Zheng L. Ishii Y. Tokunaga A. Hamashima T. Shen J. Zhao Q. L. (2010). Neuroprotective effects of PDGF against oxidative stress and the signaling pathway involved. J. Neurosci. Res. 88 (6), 1273–1284. 10.1002/jnr.22302 19998489

[B39] Zhou Y. Wu Y. Liu Y. He Z. Zou S. Wang Q. (2017). The cross-talk between autophagy and endoplasmic reticulum stress in blood-spinal cord barrier disruption after spinal cord injury. Oncotarget 8 (1), 1688–1702. 10.18632/oncotarget.13777 27926492 PMC5352089

